# Annexin A5 prevents amyloid-β-induced toxicity in choroid plexus: implication for Alzheimer’s disease

**DOI:** 10.1038/s41598-020-66177-5

**Published:** 2020-06-10

**Authors:** Fernando Bartolome, Agnieszka Krzyzanowska, Macarena de la Cueva, Consuelo Pascual, Desiree Antequera, Carlos Spuch, Alberto Villarejo-Galende, Alberto Rabano, Juan Fortea, Daniel Alcolea, Alberto Lleo, Isidro Ferrer, John Hardy, Andrey Y. Abramov, Eva Carro

**Affiliations:** 1 Networking Biomedical Research Center on Neurodegenerative Diseases (CIBERNED), Madrid, Spain; 2 Group of Neurodegenerative Diseases, Hospital 12 de Octubre Research Institute (imas12), Madrid, Spain; 3 Neuroscience Translational Group, Galicia Sur Health Research Institute, SERGAS-Universidad de Vigo; CIBERSAM, Vigo, Spain; 4 Neurology service Hospital Universitario 12 de Octubre, Madrid, Spain; 5 Department of Neuropathology and Tissue Bank, Unidad de Investigación Proyecto Alzheimer, Fundación CIEN, Instituto de Salud Carlos III, Madrid, Spain; 6 Memory Unit, Neurology Department, Hospital de la Santa Creu i Sant Pau, Barcelona, Spain; 7 Institut d’Investigacions Biomediques Sant Pau - Universitat Autònoma de Barcelona, Barcelona, Spain; 8 IDIBELL-Hospital Universitari de Bellvitge, Hospitalet de Llobregat, Hospitalet de Llobregat, Spain; 9 Universitat de Barcelona, Hospitalet de Llobregat, Hospitalet de Llobregat, Spain; 10 Department of Neurodegenerative Disease, UCL Queen Square Institute of Neurology, London, United Kingdom; 11 Department of Clinical and Movement Neurosciences, UCL Queen Square Institute of Neurology, London, United Kingdom

**Keywords:** Cell death in the nervous system, Bioenergetics, Neurological disorders

## Abstract

In Alzheimer’s disease (AD) amyloid-β (Aβ) deposits may cause impairments in choroid plexus, a specialised brain structure which forms the blood–cerebrospinal fluid (CSF) barrier. We previously carried out a mass proteomic-based study in choroid plexus from AD patients and we found several differentially regulated proteins compared with healthy subjects. One of these proteins, annexin A5, was previously demonstrated implicated in blocking Aβ-induced cytotoxicity in neuronal cell cultures. Here, we investigated the effects of annexin A5 on Aβ toxicity in choroid plexus. We used choroid plexus tissue samples and CSF from mild cognitive impairment (MCI) and AD patients to analyse Aβ accumulation, cell death and annexin A5 levels compared with control subjects. Choroid plexus cell cultures from rats were used to analyse annexin A5 effects on Aβ-induced cytotoxicity. AD choroid plexus exhibited progressive reduction of annexin A5 levels along with progressive increased Aβ accumulation and cell death as disease stage was higher. On the other hand, annexin A5 levels in CSF from patients were found progressively increased as the disease stage increased in severity. In choroid plexus primary cultures, Aβ administration reduced endogenous annexin A5 levels in a time-course dependent manner and simultaneously increased annexin A5 levels in extracellular medium. Annexin A5 addition to choroid plexus cell cultures restored the Aβ-induced impairments on autophagy flux and apoptosis in a calcium-dependent manner. We propose that annexin A5 would exert a protective role in choroid plexus and this protection is lost as Aβ accumulates with the disease progression. Then, brain protection against further toxic insults would be jeopardised.

## Introduction

Alzheimer’s disease (AD) is a progressive neurodegenerative disorder and the most common cause of dementia in elderly^1,2^. The imbalance between amyloid-β (Aβ) peptide generation from amyloid precursor protein^3^ and clearance, induces its accumulation, aggregation, and deposition in the brain, which is thought to be an early and main pathogenic event in AD^2,4^. Besides accumulation and production in specific parenchyma areas, such as hippocampus and cortex, and blood vessels^5^, Aβ also accumulates in choroid plexus^6,7^. More specifically it was demonstrated that Aβ may be additionally produced and degraded in choroid plexus along with other brain areas^8–10^. Choroid plexus is a monolayer of specialised epithelial cells in the brain ventricles forming the blood – cerebrospinal fluid (CSF) barrier. The choroid plexus main function is to produce and secrete CSF (CSF turnover) protecting therefore CSF against external toxic insults. Works carried out by Jean Marie Serot *et al*., found choroid plexus functions of secretion, synthesis, and transport were deteriorated in AD as they observed morphological modifications including epithelial atrophy, fibrosis and calcifications of stroma, and thickened basement membrane^11^. These impairments resulted in lower turnover and CSF stasis, reduced transthyretin levels, a sequestering protein synthesised by choroid plexus, and oligomerisation and subsequent accumulation of Aβ (reviewed in^12^). Also, it was demonstrated that Aβ peptides accumulation in choroid plexus is largely responsible for an increased level of oxidative stress and cell death^7,13^. A decreased activity of enzymes involved in oxidative phosphorylation^14^ and mitochondrial activity^7^, may also contribute to impair protein synthesis in choroid plexus. The observed decrease in the choroid plexus functional activity would be partially correlated with reduced protein secretion and mentioned CSF renewal, which may be involved in the initiation and progression of AD^12^. Although the detailed mechanism of Aβ-induced toxicity in choroid plexus is unclear, perturbation of Ca^2+^ homeostasis, and destabilisation of cellular metabolism by pronounced membrane permeabilisation might likely play an important role.

Annexin A5, a Ca^2+^-regulated, phospholipid-binding protein belongs to the annexins superfamily. This protein is abundantly expressed in a wide range of tissues^15^ with intra- and extracellular locations^16^. Although annexin A5 is extensively used as an indicator of early apoptosis, it was demonstrated that annexin A5 exerts protective functions including inhibition of proinflammatory response and improvement of cardiac function and survival during endotoxemia in mice^17^. Also, annexin A5 was found interacting with amyloidogenic proteins reducing its toxicity in neurodegenerative diseases and type II diabetes mellitus^18^. It was also shown annexin A5 provided protection against Aβ cytotoxicity, and it was proposed that this effect occurs by competitive interaction with phosphatidylserine (PS) on the membrane surface^19–21^.

Annexins are predominately located within the cell, where they mediate cellular processes such as exocytosis and endocytosis, membrane structure and generation of lipid rafts, but also extracellular roles such as inflammation^22,23^. Particularly, it was reported annexin A5 has antithrombotic properties, reducing vascular inflammation and improving endothelial function^24–26^. Annexin A5 was proposed as biomarker in heart injury^27^ and also in nephrotic syndrome^28^. Moreover, annexin A5 was associated with various neuropathological conditions. Levels of annexin A5 were reported reduced in CSF from Parkinson’s disease (PD) patients^29^. It was suggested that such reduction resulted as consequence of protein consumption during neuronal apoptosis^29^. Plasma levels of annexin A5 were found significantly higher in AD patients compared with healthy individuals^30^. These works suggested a defensive function for annexin A5 in these neurodegenerative diseases based on its interacting role with amyloidogenic proteins, islet amyloid polypeptides and α-synuclein inclusions by reducing the toxicity of these proteins and aggregates^18^.

Using a mass proteomic based study, we previously found changes in annexin A5 protein levels in choroid plexus from AD patients^31^. The present study was focused on examining the potential effects of annexin A5 on Aβ toxicity in choroid plexus. For that purpose, we analysed choroid plexus tissue samples and CSF from MCI and AD patients, and choroid plexus primary cultures. Here, we show annexin A5 levels in CSF were found progressively increased as the disease stage increased in severity. Simultaneously, annexin A5 levels were found decreased with the disease severity in choroid plexus, in opposite way Aβ burden and cell death. We also show that annexin A5 may play a protective role in Aβ-induced cell toxicity in choroid plexus by a Ca^2+^-dependent mechanism.

## Results

### Annexin A5 levels are increased in CSF from AD patients

**Figure 1:**
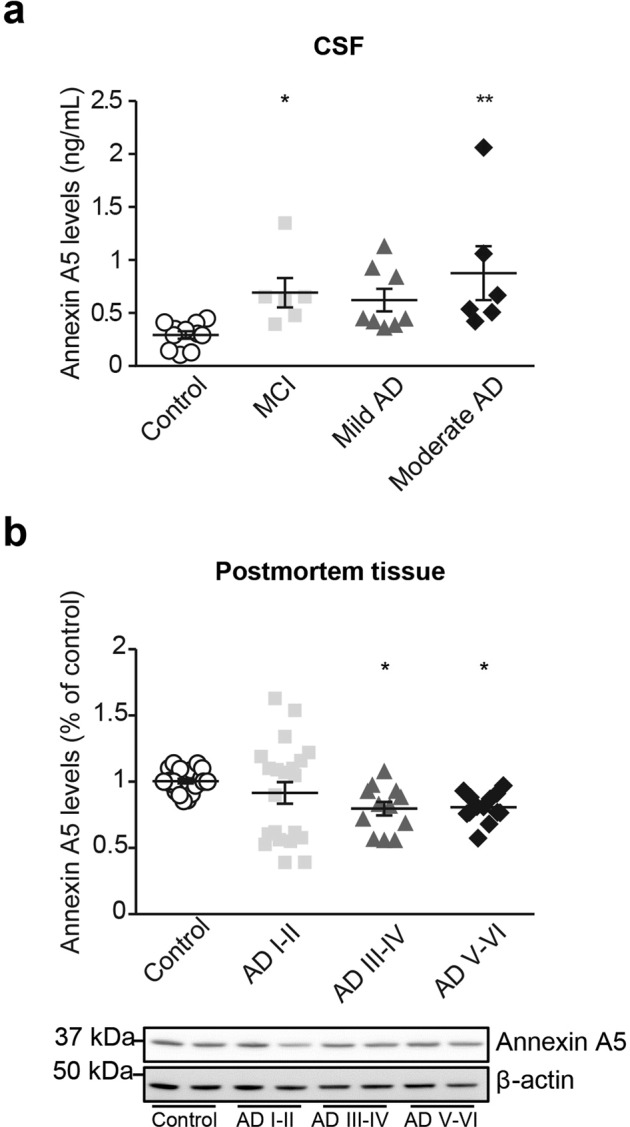
CSF and choroid plexus annexin A5 protein levels were differentially expressed in AD patients. (**a**) Scatter plot showing CSF levels of annexin A5 protein in healthy donors and clinically diagnosed MCI and AD patients determined by ELISA. Annexin A5 levels in CSF samples were found higher in MCI and AD patients compared with controls (*p < 0.05, **p < 0.01; control n = 11; MCI n = 6; Mild AD n = 8; Moderate AD n = 6). In all cases, each individual value is shown along with the mean ± SEM per group. (**b**) Scatter plot showing annexin A5 protein levels in choroid plexus from AD and healthy donors. Annexin A5 levels were significantly reduced in choroid plexus from Braak stages III/IV and V/VI compared with control samples (*p < 0.05; control n = 22; AD I-II n = 21; AD III-IV n = 12; AD V-VI n = 13). In all cases, each individual value is shown along with the mean ± SEM per group. Bottom images show representative western blots. These images were cropped from full-length blots and they are shown in the Supplementary Information section.

By doing a mass proteomic based study, we previously found annexin A5 protein levels were differentially expressed in choroid plexus from AD patients compared with healthy controls^31^. Here, we show annexin A5 levels were significantly increased in CSF from MCI and moderate AD patients determined by ELISA (Fig. 1a). We also checked by Western Blot annexin A5 levels in choroid plexus from brain donors with AD pathology and healthy subjects. Annexin A5 protein levels were significantly lower in Braak stages III/IV and V/VI compared with control samples (Fig. 1b). In contrast with our previous work^31^, by increasing the number of samples here we increased the variability losing the significance when comparing AD subjects (stages I-II) with control. When comparing last AD stages with controls, variability was reduced, and results reflect a significant reduction in annexin A5 levels in advanced AD stages compared with aged-matched control subjects. Together, these results could suggest annexin A5 levels in choroid plexus progressively are reduced starting at early AD clinical stages.

### Aβ-related choroid plexus cell death

**Figure 2:**
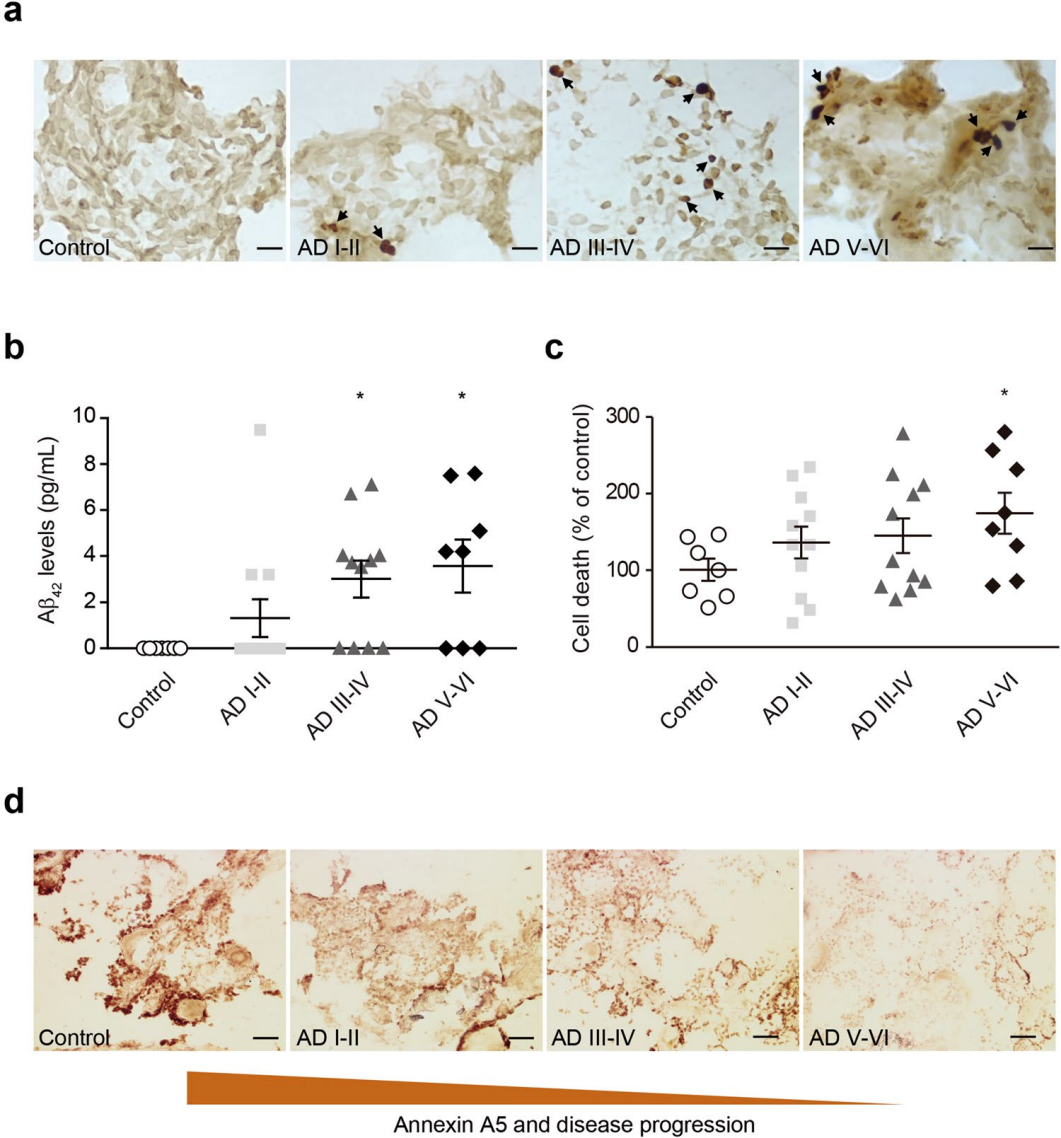
Aβ deposits and cell death in choroid plexus from AD patients. (**a**) Representative micrographs showing Aβ deposits in choroid plexus from early and advanced AD cases compared with healthy donors. Scale bar = 20 μm. (**b**) Quantified levels of Aβ burden in choroid plexus from healthy donors compared with AD subjects measured by ELISA (Cell Death Detection ELISAPLUS kit, Roche). Scatter plot reveals a progressive increase in Aβ levels in choroid plexus samples from AD subjects compared with control group (*p < 0.05; control n = 7; AD I-II n = 12; AD III-IV n = 11; AD V-VI n = 8). In all cases, each individual value is shown along with the mean ± SEM per group. (**c**) Cell death analysis in choroid plexus from control and AD patients was measured using Cell Death Detection ELISAPLUS kit. Scatter plot shows a significant increase in cell death in choroid plexus from advanced AD patients (Braak stages V–VI) compared with control subjects (*p < 0.05; control n = 7; AD I-II n = 11; AD III-IV n = 11; AD V-VI n = 8). In all cases, each individual value is shown along with the mean ± SEM per group. **(d**) Representative micrographs showing annexin A5 immunostaining in choroid plexus from early and advanced AD cases compared with healthy donors. Scale bar = 20 μm.

AD-related pathology was confirmed analysing the Aβ load in choroid plexus by immunohistochemistry using an anti-Aβ antibody (Fig. 2a). The progressive pattern of Aβ accumulation in choroid plexus was estimated using an Aβ_42_ human specific ELISA kit (Fig. 2b). Aβ_42_ burden was significantly higher at Braak stages III/IV and stages V/VI when compared with control cases (Fig. 2b). The effects of Aβ accumulation determined in choroid plexus using a Cell Death Detection ELISA kit resulted in progressive increased cell death in AD subjects that was significantly higher at Braak stages V/VI compared with healthy donors (Fig. 2c). These results were consistent with the previously reported findings showing deleterious effects of Aβ in choroid plexus from AD subjects and the AD transgenic APP/PS1 mouse model^7^. It is important to note that the observed progressive Aβ accumulation and cell death in choroid plexus match with the reduced annexin A5 levels (Fig. 1b), more evident at late AD stages (III-VI) (Fig. 2d).

### Aβ induces Annexin A5 release outside choroid plexus cells

**Figure 3:**
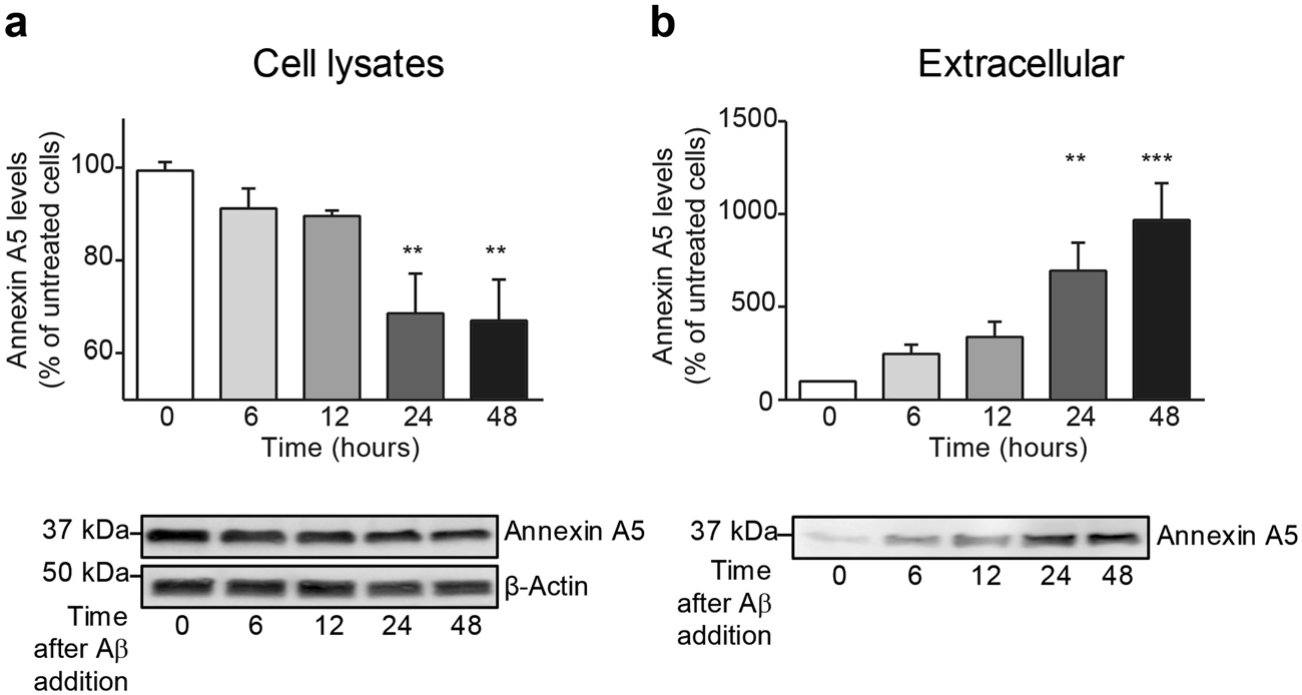
Analysis of intra- and extracellular annexin A5 levels in choroid plexus cultures after Aβ incubation. (**a**) Annexin A5 levels in epithelial choroid plexus cell cultures incubated with oligomerised Aβ_42_ for 6, 12, 24 and 48 hours determined by Western blot. Annexin A5 levels are shown as percentage of untreated cells. β-actin was used as loading control. Histogram reveals a progressive decrease in annexin A5 levels in cells incubated with Aβ_42_ already significant at 24 hours (upper panel) (**p < 0.01; n = 4). Representative bands of annexin A5 protein levels in choroid plexus cells at 0, 6, 12, 24 and 48 hours after Aβ_42_ addition (bottom panel). These images were cropped from full-length blots and they are shown in the supplementary information section. In all cases data represents mean ± SEM. **(b**) Annexin A5 levels in extracellular medium of Aβ_42_-incubated choroid plexus cells for 0, 6, 12, 24 and 48 hours. Histogram shows a progressive increase of annexin A5 levels in extracellular medium which is already significant 24 hours after Aβ_42_ incubation (upper panel) (**p < 0.01; ***p < 0.001; n = 4). Representative bands of annexin A5 protein levels in extracellular medium at 0, 6, 12, 24 and 48 hours after Aβ_42_ addition (bottom panel). This image was cropped from a full-length blot shown in the Supplementary Information section. In all cases data represents mean ± SEM.

Annexin A5 may be secreted extracellularly and increased annexin A5 levels were previously found in the supernatant of Aβ_42_-treated neuronal cells^32^. Hence, we asked if changes in annexin A5 levels between choroid plexus and CSF could be related to Aβ overload in choroid plexus. We used rat primary cultures of epithelial choroid plexus cells treated with oligomerised Aβ_42_ (10 µM) at 6, 12, 24 and 48 hours. Aβ exposure in choroid plexus cells resulted in reduced annexin A5 protein levels in a time course-dependent manner (Fig. 3a). This reduction was significant compared with untreated cells 24 and 48 hours after Aβ treatment (Fig. 3a). Conversely, Aβ induced a time course-dependent increase of annexin A5 in the extracellular medium (Fig. 3b). Such annexin A5 increase in supernatant was clearly significant 24 and 48 hours after Aβ treatment (Fig. 3b).

### Annexin A5 restores the Aβ-induced impairments on autophagy

**Figure 4:**
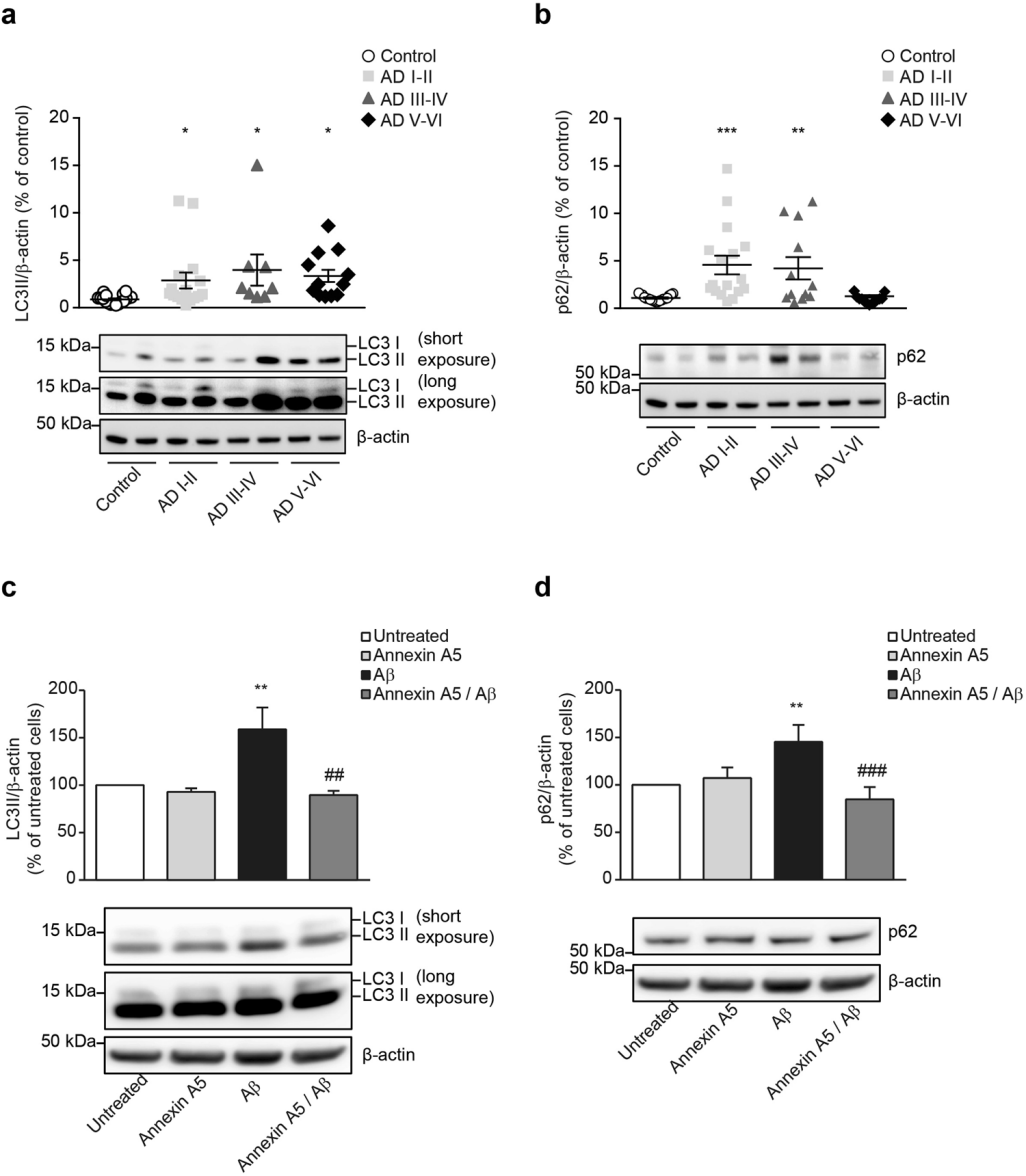
Impaired autophagy in AD choroid plexus. (**a,b**) LC3-II (**A**) and p62 (**b**) protein levels in choroid plexus from healthy donors and AD patients from I, II, III, IV, V and VI Braak stages determined by Western blot. β-actin was used as loading control and data are shown as percentage of untreated cells. (**a**) Scatter plot indicates that autophagic marker LC3-II is significantly increased at early AD stages (Braak I-II). (**b**) p62 is significantly increased at early (Braak I-II) and mid (Braak III-IV) AD stages but decreased at late AD stages (Braak V-VI). Representative Western blot showing LC3-II (**a**) and p62 (**b**) levels in choroid plexus samples from healthy donors, and AD are shown. These images were cropped from full-length blots and they are shown in the Supplementary Information section. (*p < 0.05, **p < 0.01, ***p < 0.001; control n = 19; AD I-II n = 17; AD III-IV n = 12; AD V-VI n = 13). In all cases, each individual value is shown along with the mean ± SEM per group. (**c,d**) LC3-II (**c**) and p62 (**d**) protein levels in epithelial choroid plexus cell cultures incubated with and without oligomerised Aβ_42_ (10 μM) for 24 hours under presence or absence of annexin A5 (15μg/ml). β-actin was used as loading control and data are shown as percentage of untreated cells. (**c**) Autophagic marker LC3-II is significantly increased upon Aβ_42_ incubation compared with untreated or annexin A5-treated cells. Annexin A5 co-incubation restored the Aβ_42_-increased autophagic markers levels similar to untreated cells. (**d**) Autophagic marker p62 is significantly increased upon Aβ_42_ incubation compared with untreated or annexin A5-treated cells. Annexin A5 co-incubation restored the Aβ_42_-increased autophagic markers levels similar to untreated cells. Representative Western blot showing LC3II (**c**) and p62 (**d**) levels in epithelial choroid plexus cell cultures are shown. These images were cropped from full-length blots and they are shown in the Supplementary Information section. (**p < 0.01 versus untreated cells; ^##^*p* < 0.01, ^###^*p* < 0.001 versus Aβ_42_-treated cells; n = 6). In all cases data represents mean ± SEM.

A number of studies demonstrate autophagy is disrupted at one point in the progression of AD^33,34^. This pathway may degrade accumulated aberrant proteins and peptides such as Aβ. Results above could suggest that annexin A5 may be beneficial in choroid plexus from AD patients; therefore we investigated the effect of annexin A5 on autophagy. Analysis of autophagy markers in postmortem tissue from AD and healthy donors by immunoblotting, showed significantly increased LC3-II levels in choroid plexus from all AD stages including early AD (Fig. 4a) and significantly increased p62 levels at Braak stages I-IV (Fig. 4b). These results may suggest both, increased autophagy induction and disrupted autophagosome – lysosome fusion^35,36^. Then we investigated annexin A5 effects on Aβ-disrupted autophagy flux in choroid plexus cultures from rats. Cells were treated with and without annexin A5 for 1 hour and then exposed to oligomerised Aβ_42_ to avoid extracellular Aβ sequestering by annexin A5, thereby preventing the toxicity. Immunoblotting revealed that Aβ_42_ exposure resulted in significantly elevated LC3-II and p62 levels compared with untreated cells suggesting autophagosome accumulation (Fig. 4c,d, respectively). Annexin A5 treatment in cells restored the LC3-II and p62 levels being similar to the obtained values in untreated or cells treated with annexin A5 only (Fig. 4c,d, respectively). In summary, the above results could indicate annexin A5 abolished the Aβ-induced impairments on autophagy in choroid plexus cells.

### Annexin A5 protects against Aβ-induced apoptosis

**Figure 5:**
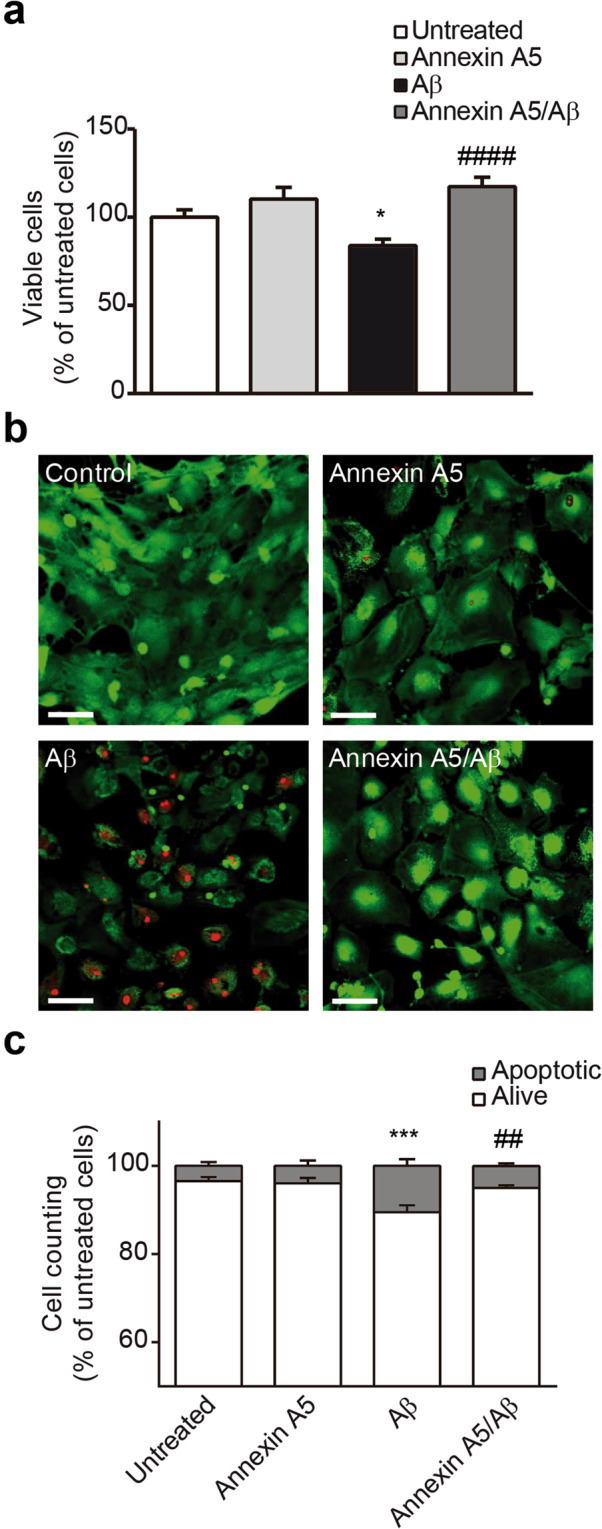
Annexin A5 restores the Aβ-induced cell viability reduction and apoptosis. (**a**) Epithelial choroid plexus cell viability after incubation with and without oligomerised Aβ_42_ (10 µM) for 48 hours in absence or presence of annexin A5. A significant decrease in cellular viability was found in Aβ_42_ incubated cells compared with untreated cells or cells treated with annexin A5 only. Co-administration of annexin A5 (15 µl/ml) and Aβ_42_ (10 µM) restored cell viability in choroid plexus epithelial cell cultures (*p < 0.05 versus untreated cells; ^####^*p* < 0.0001 versus Aβ_42_-treated cells; n = 7). In all cases, data represents mean ± SEM. (**b**) Apoptotic cell death was analysed using the LIVE/DEAD Viability/Cytotoxicity Kit (Molecular Probes). Fluorescent images of epithelial choroid plexus cell culture show living cells (green) and apoptotic cells (red). Cells were incubated with and without oligomerised Aβ_42_ (10 µM), in absence and presence of annexin A5 (15 µl/ml). Scale bar = 44 μm. (**c**) Counting alive (green) and apoptotic (red) cells shows that Aβ_42_ incubation induced a significant increase of apoptotic cells compared with untreated or annexin A5 treated cells. Co-administration annexin A5 and Aβ_42_ reduced the number of apoptotic cells (***p < 0.001 versus untreated cells; ^##^*p* < 0.01 versus Aβ_42_-treated cells; n = 4). In all cases, data represents mean ± SEM.

It was previously shown annexin A5 inhibited Aβ toxicity in neuronal cell cultures^16,17,19,20,34,37^. We further investigated annexin A5 effect on Aβ-induced toxicity in choroid plexus cells. As Aβ-induced autophagy may precede Aβ-induced toxicity we tested the biological role of annexin A5 on cell viability carrying out bioactivity assays with choroid plexus cell cultures. Cells were previously treated with and without annexin A5 for 1 hour and then exposed to oligomerised Aβ_42_ (10 µM) for 48 hours (Fig. 5). Aβ_42_ induced 25% decrease in cell viability in choroid plexus cultures, as assessed by cell counting kit (CCK) assay, compared with control (Fig. 5a). Annexin A5 completely prevented cell loss when was added prior to Aβ_42_ (Fig. 5a). The reduction in cell viability on choroid plexus cells and the protective role of annexin A5 was studied analysing apoptotic cell death using the LIVE/DEAD Viability/Cytotoxicity Kit (Fig. 5b,c). 48 hours after incubation with Aβ_42_ (10 µM), a significant increase of apoptotic cell death in choroid plexus cultures was found (Fig. 5b,c). The presence of annexin A5 attenuated the Aβ_42_-induced apoptosis, recovering the amount of living cells and reducing the cell loss (Fig. 5b,c).

### Annexin A5 attenuates Aβ-induced impairments on mitochondrial function and [Ca^2+^] homeostasis in choroid plexus epithelial cells

**Figure 6:**
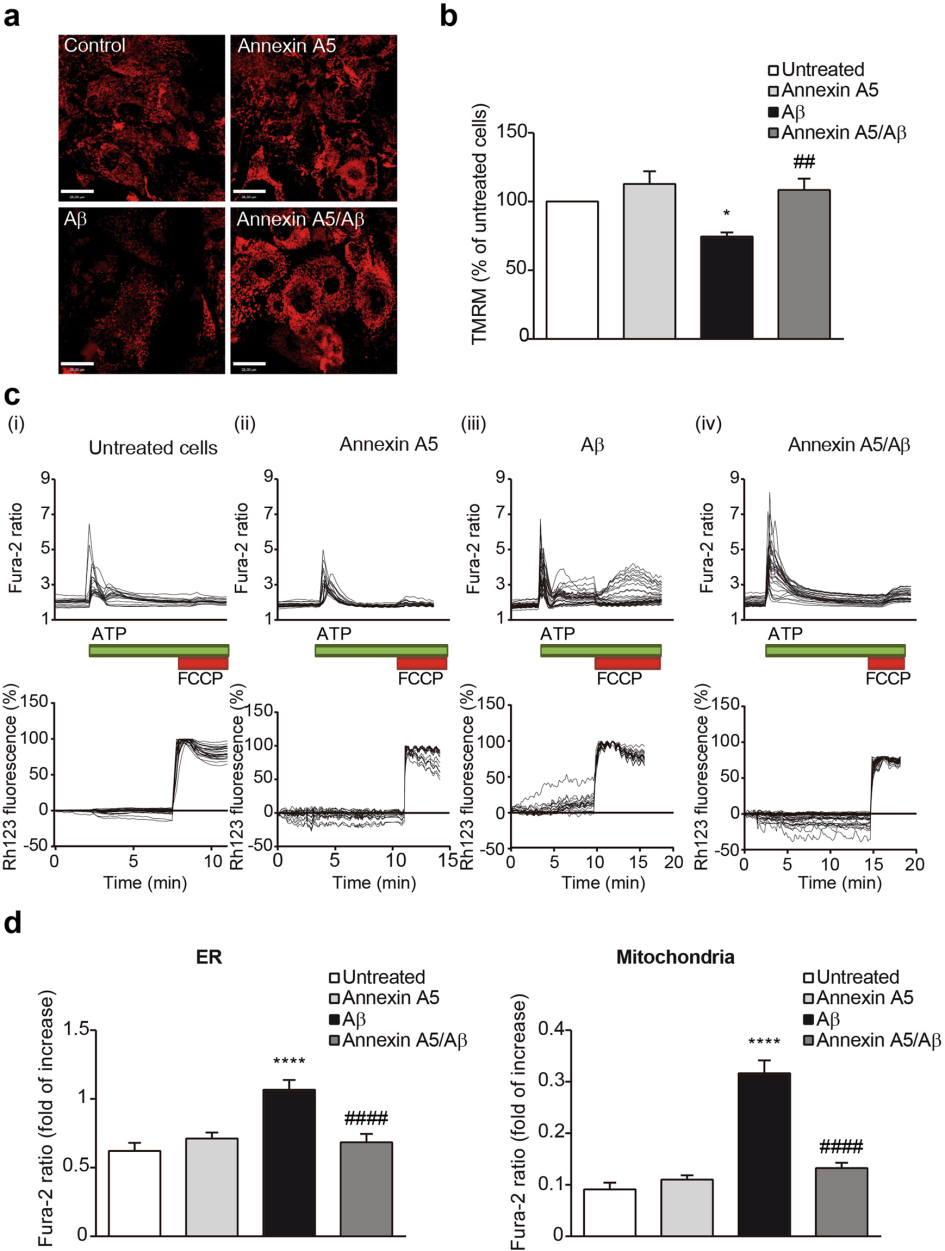
Annexin A5 restores mitochondrial depolarisation in Aβ-treated choroid plexus cultures in a Ca^2+^-dependent manner. (**a**) Representative images of tetramethyl-rhodamine methylester (TMRM) fluorescence used in redistribution mode (40 nM) in choroid plexus cultures incubated with and without oligomerised Aβ_42_ (10 µM), in absence and presence of annexin A5 (15 µl/ml). **(b**) Annexin A5 restored the Aβ-induced mitochondrial depolarisation. Data were normalised to untreated cells and are represented as mean ± SEM from at least three independent experiments. (*p < 0.05 versus untreated cells; ^##^*p* < 0.01 versus Aβ_42_-treated cells; n = 4). (**c**) Traces showing changes-over time in fura-2 (upper panels) and simultaneous rhodamine 123 (Rh123) (bottom panels) fluorescence in choroid plexus epithelial cells upon physiological Ca^2+^ stimuli. Physiological Ca^2+^ was induced by addition of ATP (100 μM). Following Ca^2+^ release from ER, FCCP (1 μM) was added to obtain the maximal mitochondrial depolarisation allowing mitochondrial Ca^2+^ release. Upon stimulation of choroid plexus cells with ATP, Ca^2+^ stored in the ER was released and profound mitochondrial depolarisation was found in Aβ-incubated cells as shown by the increase in the Rh123 signal (iii). This effect was not observed in untreated cells (i) or annexin A5-treated cells only (ii). Annexin A5 treatment prevented the mitochondrial depolarisation when Ca^2+^ stimulus was added (iv). (**d**) Histograms showing ER Ca^2+^ (left panel) and mitochondrial Ca^2+^ levels (right panel) after addition of ATP and FCCP respectively as explained above. ER Ca^2+^ in choroid plexus cells incubated with Aβ were higher compared with untreated cells or annexin A5-treated cells only. Annexin A5 co-treatment restored the Aβ-induced increase of ER Ca^2+^ levels. Mitochondrial Ca^2+^ in Aβ-incubated choroid plexus cells was significantly higher compared with untreated cells or annexin A5-treated cells only. These levels were restored upon co-incubation with annexin A5. Data were normalised to untreated cells and are represented as mean ± SEM from at least three independent experiments. (****p < 0.0001 versus untreated cells; ^####^*p* < 0.0001 versus Aβ_42_-treated cells; n = 4).

Both, apoptosis and autophagy are two cellular processes closely linked to mitochondria and mitochondrial health and function is reflected in the ΔΨ_m_. By using the fluorescent indicator TMRM, we analysed the ΔΨ_m_ (Fig. 6a). This dye accumulates in active mitochondria with intact membrane potentials, therefore the more fluorescence, the more mitochondrial health and quality. A significant decrease in ΔΨ_m_ was observed in Aβ-exposed choroid plexus cells reducing the TMRM signal to 74 ± 3% compared with either untreated or cells treated with annexin A5 (Fig. 6b). Treatment of Aβ-exposed choroid plexus cells with annexin A5 prevented the reduction in the ΔΨ_m_ as TMRM signal showed similar values to untreated cells or cells with annexin A5 (Fig. 6b). Mitochondria are master regulators of Ca^2+^ homeostasis^38^ that simultaneously is linked to autophagy and apoptotic cell death regulation. Because annexin A5 is a Ca^2+^ binding protein we hypothesised that the protective role of annexin A5 could be related to its Ca^2+^ binding capacity. This could be supported by findings in which was demonstrated Aβ-induced toxicity in neurons involved perturbation of Ca^2+^ homeostasis^39^. We analysed Ca^2+^ flux upon physiological Ca^2+^ stimuli in choroid plexus cells incubated for 24 hours with oligomerised Aβ_42_ in absence or presence of annexin A5 (Fig. 6c). Ca^2+^ signal was evaluated using fura-2. Purinergic receptors in epithelial choroid plexus cells were stimulated with ATP allowing the massive release of Ca^2+^ from the endoplasmic reticulum (ER) to the cytosol which was ratiometrically quantified (Fig. 6c, top panels). After recovery, mitochondrial Ca^2+^ uptake was analysed by adding the uncoupler FCCP and the amount of Ca^2+^ was quantified (Fig. 6c, top panels). Upon physiological Ca^2+^ stimuli, changes in ΔΨ_m_ were simultaneously analysed using Rh123 and the fluorescence was also quantified (dequench mode, Fig. 6c, bottom panels). ATP application to Aβ-treated choroid plexus cells was associated to loss of ΔΨ_m_ as Rh123 signal increased by 23% before FCCP addition. Annexin A5 treatment to Aβ-incubated choroid plexus cells prevented the mitochondrial depolarisation as Rh123 fluorescence did not increase after ATP addition. We found that Aβ_42_ incubation resulted in a significantly higher Ca^2+^ release from the ER in response to ATP in choroid plexus cells (Fig. 6d, left panel). Simultaneous incubation with Aβ and annexin A5 restored the ATP-induced Ca^2+^ release from the ER in choroid plexus cells resulting in a smaller Ca^2+^ signal similar to the obtained in untreated or only annexin A5 treated cells (Fig. 6d, left panel). Mitochondrial Ca^2+^ uptake after FCCP addition was also higher in Aβ treated cells compared with untreated or only annexin A5 treated cells (Fig. 6d, right panel). Treatment of choroid plexus cells with annexin A5 under Aβ presence restored the mitochondrial Ca^2+^ uptake reaching similar levels of the untreated cell or cells treated with annexin A5 (Fig. 6d, right panel). Together, these results confirmed annexin A5 exerts its protective role on Aβ-induced autophagy and apoptotic cell death in choroid plexus in a Ca^2+^-dependent manner controlled by mitochondria.

## Discusion

Annexins are widely distributed among tissues and their ubiquitous distribution suggests they have important roles in cell biology. In particular, annexin A5 has been found exerting anti-inflammatory, anticoagulant and anti-apoptotic functions^40–46^. In endothelial cells, annexin A5 was able to protect against vascular inflammation contributing to protect barrier integrity^25^. Expression of other annexins, annexin A1, A4 and A6, was described in choroid plexus^47,48^, but little is known about expression and role of annexin A5 in this brain structure. Here we report increased annexin A5 levels in CSF from clinically diagnosed MCI and AD patients and simultaneous reduced annexin A5 levels in choroid plexus from AD brains. These findings are accompanied by increased Aβ overload in choroid plexus related to the disease severity and significantly altered autophagic flux and increased cell death at late AD stages. *In vitro*, we also demonstrate that annexin A5 extracellular release in choroid plexus cells could be Aβ-overload dependent. We propose that Aβ in choroid plexus may induce increased autophagosome accumulation. Such effects result finally in increased cell death and annexin A5 down-regulation. On the other hand, annexin A5 protects against Aβ toxicity restoring the Aβ-induced Ca^2+^ homeostasis dysregulation linked to the mitochondrial health and function. Together, our findings suggest that annexin A5 plays a crucial role in maintaining physiological protection of blood-CSF barrier that is impaired in AD.

The higher levels of annexin A5 found in CSF from MCI and AD subjects compared with healthy donors suggests that secreted annexin A5 may indicate early changes in choroid plexus from these patients. Increased annexin A5 levels were also found in plasma from AD patients and the AD transgenic mouse model Tg2576^32^. We suggest that choroid plexus epithelial cells contribute to increase the annexin A5 levels in CSF along with other sources as neurons. Our results indicate CSF levels of annexin A5 become increased even at early stages of disease, including MCI, when Aβ accumulation in the brain becomes evident. This is consistent with other findings observed in neuronal cell cultures, describing increased annexin A5 levels in the supernatant of Aβ_42_-treated cells^32^.

Aβ deposition in brains of AD patients agrees with the hypothesis suggesting Aβ accumulation as the main cause for neuronal cell death^1,5,49^. Exposure of cells to Aβ peptides cause typical apoptotic cell death^50,51^, although lower Aβ concentrations appear to only affect expression of neuronal apoptotic genes, without directly causing apoptosis^52,53^. Here we show that Aβ accumulation in AD is also present in choroid plexus. This observation was found gradually increasing as the disease progresses and is consistent with the increased cell death observed in choroid plexus from AD subjects. We demonstrate Aβ overload in choroid plexus cultures induced annexin A5 reduction in cells and subsequently increased annexin A5 in extracellular medium. Annexin A5 release outside cells occurs from the cellular apical compartment as has been demonstrated in epithelial cell cultures^46,54^. Upon Ca^2+^ stimulation, cytoplasmic annexin A5 is translocated to the plasma membrane while nuclear annexin A5 is moved to the nuclear membrane, in all cases binding to phosphatidylserine (PS)^55^. As Aβ also binds PS, both annexin A5 and Aβ could exert competitive interaction to each other. Reduced levels of intracellular annexin A5 result in lower competitive inhibition with Aβ at PS interaction site leading to increasing Aβ-induced cytotoxicity^19^. We may suggest Aβ overload in patient choroid plexus could induce annexin A5 drain from these cells, hence contributing to the observed increased levels in CSF.

Our experiments show that annexin A5 restores the Aβ-induced autophagic flux impairments in choroid plexus cells reducing the Aβ-increased levels of LC3-II and p62. Previous works showed elevated LC3-II and p62 levels in brains from AD patients at different stages of disease^56,57^. Here we found an increase in these autophagy markers in human choroid plexus suggesting that such increase is due to Aβ overload. In support of this observation several works showed similar effects increasing autophagy markers after Aβ incubation in human neuroblastoma H4 cells^58^ and in cells overexpressing mutant forms of *PSEN2* and *APP*, both linked to Aβ overproduction in AD familial cases^59,60^. Therefore, we may suggest an induction of autophagy is triggered in choroid plexus due to Aβ overload. Then autophagosomes containing Aβ cannot be fused with lysosomes resulting in their accumulation and subsequent increased levels of autophagic markers LC3-II and p62. Apart of the mentioned cellular models^59,60^ this dual effect has been also observed in AD brains showing high autophagy flux. Such increased flux would try to maintain low Aβ levels through autophagic clearance but this is followed by impaired autolysosomal proteolysis^33,34,57,61–63^. We found that annexin A5 treatment restored the levels of autophagy markers to similar values that those obtained in untreated cells. We suggest the annexin A5 effect on autophagy may work enhancing autophagosome – lysosomes fusion allowing lysosomal cargo degradation. This agrees with other observations where annexin A5 was proposed stimulating autophagy^64^. In this work, authors showed that overexpression of annexin A5 in HEK293T cells decreased LC3-II levels. They proposed such decrease was produced by the annexin A5 effect of increasing the autophagosome conversion into autolysosomes and enhancing lysosomal proteolysis^64^.

Linked to the protective role on autophagy could be the observation that annexin A5 reduced the number of Aβ-induced apoptotic cells. The idea of Aβ-induced cell death is preceded by impaired autophagy was also showed by other authors^65,66^. Further analysis about this connection was discussed by Nixon and Yang where they proposed defects in autophagy promoting apoptotic cell death in neurodegenerative diseases^67^. This is consistent with our results as we observed a reduction in autophagy markers and apoptotic cell death when rat primary cultures of epithelial choroid plexus cells were incubated with Aβ in presence of annexin A5. Both, stimulation of autophagy and apoptotic cell death are tightly regulated by intracellular Ca^2+^ levels^68,69^. On this regard, mitochondria play an important role in cells as they control bioenergetics, Ca^2+^ homeostasis and apoptotic cell death. Failures in this regulation have been recognised in neurodegenerative disorders. Here, we show that annexin A5 restored the Aβ-induced mitochondrial depolarisation reflected by a reduction in the mitochondrial membrane potential which mirrors the mitochondrial health and function. Therefore, mitochondrial depolarisation may indicate altered Ca^2+^ homeostasis regulation. Indeed, our work show Aβ impaired Ca^2+^ homeostasis, and annexin A5 prevented this effects. Aβ exposure made the choroid plexus cells to increase the ER Ca^2+^ stores. Then, by inducing Ca^2+^ signal, mitochondrial Ca^2+^ uptake was higher. Both effects were restored by annexin A5 incubation. It has been shown Aβ affects intracellular Ca^2+^ concentration by generating Ca^2+^ permeable channels in plasma membrane of neurons and astrocytes^70,71^. Works carried out in neuronal cells showed Aβ exposure induced Ca^2+^ homeostasis impairments^72–75^. *In vivo*, elevated Ca^2+^ levels in neuronal cytoplasm were linked to Aβ_42_ accumulation^76^. The observed protective role of annexin A5 in the Aβ-induced autophagy impairments and cell death could be related to its affinity binding preferentially acidic phospholipids in a Ca^2+^ dependent manner^22^. This could be in line with the observed annexin A5 ability to interact with membrane PS. Through this interaction, it was found that annexin A5 protected neuronal cells against Aβ toxicity by competitive inhibition as both, Aβ oligomers and annexin A5 showed binding affinity to PS in the cell membrane in a Ca^2+^-dependent manner^19,20^. In a different scenario, we may also explain the protective role of annexin A5 on Aβ-induced toxicity by its Ca^2+^ chelating capacity decreasing the intracellular Ca^2+^ concentration. All these observations are consistent with our findings in which we found annexin A5 could have a potential beneficial effect on choroid plexus and such effect is lost in AD along with progressive increased Aβ accumulation. This beneficial effect of annexin A5 has been proved in other neurodegenerative disorders showing its interaction ability with amyloidogenic proteins^18^.

In summary, we show that levels of annexin A5 in choroid plexus from AD are reduced in late stages of disease, accompanied by high Aβ levels and cell death and simultaneous increased levels of annexin A5 in CSF. The data presented here indicate that annexin A5 protects choroid plexus cells from Aβ-induced autophagic impairments and apoptosis by a Ca^2+^-dependent mechanism under mitochondrial control.

## Material and Methods

All methods were performed following the relevant guidelines and regulations approved by the local ethical review committee from the Hospital 12 de Octubre Research Institute. Other review committees partially involved in the present project were the following: 12 de Octubre Hospital, (Madrid, Spain) and Hospital de la Santa Creu i Sant Pau (Barcelona, Spain) review committees for research related to CSF samples; The Institute of Neuropathology Brain Bank IDIBELL-Hospital Universitari de Bellvitge (Hospitalet de Llobregat, Spain) review committee, The Netherlands Brain Bank (NBB) (Amsterdam, The Nederlands) review committee and Banco de Tejidos, Fundación CIEN (Centro de Investigación de Enfermedades Neurológicas, Madrid. Spain) review committee for research involving the use of choroid plexus samples from human donors. Informed consent was obtained from all participants and/or their legal guardians.

### CSF collection

CSF samples (10 ml per subject) from 26 mild cognitive impairment (MCI), 20 mild AD, 20 moderate AD, and 26 healthy control subjects were collected by lumbar puncture upon informed patient consent. All samples were centrifuged at 3000 rpm at 4 °C for 10 min to remove any cell and debris, aliquoted in small volumes and stored in low bind polypropylene tubes at −80 °C. Samples were obtained from the Hospital 12 de Octubre Neurology Service (Madrid, Spain), and Hospital de la Santa Creu i Sant Pau Neurology Service (Barcelona, Spain). Subjects were clinically diagnosed as probably having AD according to the National Institute on Aging and Alzheimer’s Association (NIA-AA) guidelines^77^. MCI was diagnosed in patients with cognitive impairment that did not fulfil the criteria for dementia^78,79^. All subjects had mini-mental state examination (MMSE) scores available. Inclusion criteria for cognitively normal older individual subjects were MMSE scores 24–30, no history or clinical signs of neurological or psychiatric disease or cognitive symptoms^80^. Subjects’ consent was obtained according to Declaration of Helsinki, and approval came from the research ethics committee of each responsible institution. Written informed consent was given from all participants or representatives. Subject demographic and clinical characteristics are listed in Table 1.

**Table 1:** Demographic and clinical characteristics of CSF donors.

	Control	MCI	Mild AD	Mod AD	p-value
	(n = 26)	(n = 26)	(n = 20)	(n = 20)	
Age, mean (SD)	70.9 (9.2)	70.7 (7.3)	76.6 (6.5)	78.3 (3.6)	0.01
Sex female, n (%)	14 (53.8%)	15 (57.7%)	11 (55.0%)	10 (50.0%)	ns
MMSE, mean (SD)	29.0 (1.2)	24.5 (3.4)	19.3 (4.4)	12.3 (5.2)	<0.001
CDR	0	0.5	1	2.3	<0.001
ApoE ε4, %	0%	41.6%	46.1%	40.2%	0.01
CSF Aβ_42_, mean (sem)	885.2 (105.2)	457.3 (53.0)***	513.8 (95.9)**	339.1 (46.2)***	<0.001
CSF t-tau, mean (sem)	394.5 (54.6)	534.8 (123.5)	690.4 (73.4)*	982.8 (105.4)**	0.01

MCI: mild cognitive impairment; AD: Alzheimer’s disease; Mod: moderate-severe; SD: standard deviation; MMSE: Minimental Status Examination; ns: non-significant. # p-value indicates statistical differences between all groups; *p < 0.05, **p < 0.01, ***p < 0.001, versus control group.

### Tissue samples

Post-mortem choroid plexus tissue was obtained from brain donors diagnosed with AD and control individuals. Frozen samples were supplied by the Institute of Neuropathology Brain Bank IDIBELL-Hospital Universitari de Bellvitge (Hospitalet de Llobregat, Spain), The Netherlands Brain Bank (NBB) (Amsterdam, The Nederlands), and Banco de Tejidos, Fundación CIEN (Centro de Investigación de Enfermedades Neurológicas, Madrid. Spain). In all cases, post-mortem delays before sampling was between 3–8 hours. Subjects’ consent was obtained according to the Declaration of Helsinki, and approval came from the research ethics committee of each responsible institution. For all cases, written informed consent is available. Subjects were selected on the basis of post-mortem diagnosis of AD according to neurofibrillary pathology and Aβ plaques. Control cases were considered those without neurological symptoms and lesions in the neuropathological examination. A total of 70 samples were categorised into four groups, as presented in Table 2.

**Table 2:** Demographic and clinical characteristics of choroid plexus donors.

	Control	AD I-II	AD III-IV	AD V-VI	p-value
	(n = 22)	(n = 21)	(n = 14)	(n = 13)	
Age, mean (SD)	71.2 (10.0)	69.4 (9.9)	78.9 (7.7)	78.3 (6.6)	<0.001
Sex female, n (%)	9 (40.9%)	7 (33.3%)	6 (42.9%)	6 (46.2%)	ns

AD I-VI: Alzheimer disease-related changes, stages of Braak and Braak; SD: standard deviation; ns: non-significant.

### Choroid plexus cultures

Choroid plexus epithelial cell cultures were prepared as described previously^81^. Choroid plexus were dissected from 3–5-day-old Wistar rats (Charles River, MA, USA). ARRIVA guidelines for the care and use of animals were followed. Once disaggregated, choroid plexus epithelial cells were seeded at a 4 × 10^4^ cells/cm^2^ density on laminin-coated plates and maintained in Dulbecco’s modified Eagle’s medium (DMEM, Thermo Fisher Scientific, MA, USA) supplemented with 10% (v/v) fetal bovine serum (FBS; Thermo Fisher Scientific, MA, USA), 2 mM L-glutamine and 1% penicillin/streptomycin (Thermo Fisher Scientific, MA, USA) at 37 °C and 5% CO2. After 5–7 days on culture cells reached confluence and they were serum-deprived for 2 hours and oligomerised Aβ_42_ (10 µM; AnaSpec, Inc., San Jose, CA) was added. In the experiments with annexin A5 (15 µl/ml; Sigma-Aldrich, St. Louis, USA), this drug was added 1 hour before oligomerised Aβ_42_, to allow annexin A5 enter into the cells and avoiding extracellular sequestering of oligomerised Aβ_42_. Aβ_42_ stock was previously dissolved in acetic acid 0.1 M. Oligomeric Aβ_42_ was prepared by incubating a volume of stock solution in DMEM at 4 °C for 24 hours prior addition to the cell cultures as previously described^82^.

### Immunohistochemistry

Human choroid plexus tissue was fixed for 24 hours in 4% paraformaldehyde (PFA) by immersion. Then, choroid plexus samples were OCT embedded and stored at −80 °C for subsequent cryostat sectioning (Leica, Wetzlar, Germany). 20 µm thick sections were processed free-floating for immunohistochemistry. To carry out immunohistochemistry for Aβ deposits, brain slices were 20 min pre-incubated with 88% formic acid and immunolabeled with mouse anti-Aβ antibody (1:500, MBL, Nagoya, Japan). After overnight incubation, primary antibody staining was revealed using the avidin-biotin complex method (VECTASTAIN Elite ABC Kit, Vector Laboratories, Burlingame, CA) and 3,3′-diaminobenzidine chromogenic reaction (Vector Laboratories, Inc). Images were captured using a light microscope (Zeiss microscope; Carl Zeiss Microimaging, GmbH, Oberkochen, Germany).

### ELISA

Levels of human endogenous Aβ_42_ in choroid plexus fractions from human subjects were determined with human specific ELISA kit (Innotest β-amyloid_1–42,_ Innogenetics, Ghent, Belgium), according to the manufacturer’s instructions. Prior to tissue homogenisation, endothelial vessels were removed in order to have pure choroid plexus epithelium. Obtained data were normalised to protein content.

DNA fragmentation undergoing apoptosis was detected in choroid plexus tissue from human subjects with a Cell Death Detection ELISAPLUS kit (Roche, Basel, Switzerland). According to the manufacturer’s instructions, unfixed frozen tissue was incubated with incubation buffer (lysis buffer provided in the kit) and tissue lysates were transferred to a streptavidin-coated 96 well microplate and allowing nucleosomes to interact with monoclonal antibodies: antihistone (biotin-labeled) and anti-DNA (peroxidase-conjugated). Finally, the amount of coloured product was determined using a microplate reader.

Annexin A5 levels in CSF from human subjects were determined with specific human ELISA kit (Abcam, Cambridge, UK). Briefly, CSF samples were added to the pre-coated wells, followed by the antibody mix. Finally, the signal intensity was measured at 450 nm using a microplate reader.

### Western blot

Conditional medium from choroid plexus cultures was collected and protease and phosphatase inhibitor cocktails (Roche, Basel, Switzerland) were added. Choroid plexus tissue and cultured choroid plexus epithelial cells were homogenised in lysis buffer (50 mM Tris/HCl buffer, pH 7.4 containing 2 mM EDTA, 0.2% Nonidet P-40, 1 mM PMSF, protease and phosphatase inhibitor cocktails – Roche, Basel, Switzerland), and centrifuged for 10 min at 14000 *rpm*. The supernatant was recovered and stored at −80 °C. Protein content from cell lysates, and extracellular medium was determined with the BCA method (Thermo Fisher Scientific, MA, USA). Equal amount of protein (20 µg per lane) were separated by SDS-PAGE (4–12%) and transferred onto polyvinylidene difluoride (PVDF) membranes (Millipore, MA, USA). For human choroid plexus tissue homogenates 25 µg of protein were loaded. Non-specific bindings were blocked by incubation in 5% non-fat milk in Tris-buffered saline (100 mM NaCl, 10 mM Tris, pH 7.4) containing 0.1% Tween (TTBS) for 1 hour at room temperature. Afterwards, membranes were 4 °C incubated overnight with mouse anti-annexin A5 antibody (ab54775, 1:1000; Abcam, Cambridge, UK), rabbit monoclonal anti-p62 antibody (ab91526, 1:20000; Abcam, Cambridge, UK), and rabbit polyclonal anti-LC3 antibody (NB100–2220, 1:1000; Novus, CO, USA). Protein loading was monitored using a mouse monoclonal antibody against β-actin (A1978, 1:10000; Sigma-Aldrich, St. Louis, USA). Membranes were then incubated for 1 hour in the appropriate horseradish peroxidase-conjugated secondary antibodies (1:2000; Dako, CA, USA), and immunocomplexes were revealed by an enhanced chemiluminescence reagent (ECL Clarity; Bio Rad, CA, USA). Densitometric quantification was carried out with Image Studio Lite 5.0 software (Li-COR Biosciences, NE, USA). Protein bands were normalised to β-actin levels and expressed as percentage of the control group.

### Bioactivity and cell death quantification

*In vitro*, cell viability was assessed using Cell Counting Kit-8 (CCK-8 assay, Sigma, St. Louis, USA) according manufacturer’s instructions. Briefly, primary cultures of choroid plexus epithelial cells were incubated 48 hours with and without oligomerised Aβ_42_ (10 µM) and treated with or without annexin A5 (15 µl/ml). Then, CCK-8 solution was added, and absorbance was measured 1 hour later at 450 nm using a microplate reader.

Cell death was assessed 48 hours after incubation with and without both, oligomerised Aβ_42_ (10 µM) and annexin A5 (15 µl/ml) using the LIVE/DEAD Viability/Cytotoxicity Kit (Molecular Probes, Thermo Fisher Scientific, MA, USA). Fixed cells were labelled with propidium iodide. Healthy cells were recognised by their morphology and green staining, whereas propidium iodide-positive red cells (with condensed choromatin) were scored as apoptotic. Resulting images were taken using a fluorescence microscopy (Zeiss microscope; Carl Zeiss Microimaging, GmbH, Oberkochen, Germany) at a magnification of 40×. Fluorescence was quantified using the Volocity software (PerkinElmer, Waltham, MA, USA).

### Measurement of mitochondrial membrane potential (ΔΨ_**m**_)

ΔΨ_m_ was measured as was described previously^83^. Briefly, cells plated on 25 mm laminin-coated coverslips and loaded with 40 nM tetramethyl-rhodamine methyl ester (TMRM) in a HEPES-buffered salt solution (HBSS) (composed of 156 mM NaCl, 3 mM KCl, 2 mM MgSO_4_, 1.25 mM KH_2_PO_4_, 2 mM CaCl_2_, 10 mM glucose and 10 mM HEPES; pH adjusted to 7.35 with NaOH) for 40 min at room temperature and keeping the dye present in the chamber at the time of recording. TMRM is a cell-permeant fluorescent dye used in the redistribution mode to assess ΔΨ_m_, and therefore, a reduction in TMRM fluorescence represents mitochondrial depolarisation. Confocal images were obtained using a Zeiss 510 microscope equipped with META detection system (Zeiss, Oberkochen, Germany) and × 40 oil immersion objective. Excitation wavelength for TMRM was 560 nm and emission was detected above 580 nm. Z-stack images were obtained and analysed using the Volocity software (PerkinElmer, Waltham, MA, USA) and TMRM values for untreated cells were set to 100%. TMRM values for Aβ_42_ and/or annexin A5 treated cells were expressed relative to untreated cells.

### Imaging of [Ca^2+^]_c_

For cytosolic [Ca^2+^] ([Ca^2+^]_c_) measurements cells were plated on 25 mm laminin-coated coverslips and maintained in DMEM supplemented with 10% (v/v) FBS, 2 mM L-glutamine and 1% penicillin/streptomycin at 37 °C and 5% CO2. After 5–7 days on culture, media was replaced with HBSS (156 mM NaCl, 3 mM KCl, 2 mM MgSO_4_, 1.25 mM KH_2_PO_4_, 2 mM CaCl_2_, 10 mM glucose and 10 mM HEPES, pH adjusted to 7.35) and cells were loaded with fura-2 AM (5 µM; Molecular Probes, Thermo Fisher Scientific, MA, USA) and 0.005% pluronic for 30 minutes. For simultaneous measurement of [Ca^2+^]_c_ and ΔΨ_m_, rhodamine123 (Rh123, 10 μM; Molecular Probes, Thermo Fisher Scientific, MA, USA) was added into the cultures during the last 15 min of the fura-2 loading period, and cells were then washed. We stimulated P2Y receptors from choroid plexus cells with 100 μM ATP and we added 1 µM carbonyl cyanide-4-(trifluoromethoxy) phenylhydrazone (FCCP) to depolarise mitochondria. Fluorescence measurements were obtained on an epifluorescence inverted microscope equipped with a × 20 fluorite objective. Simultaneous [Ca^2+^]_*c*_ and ΔΨ_m_ were monitored in single cells using excitation light provided by a Xenon arc lamp, the beam passing sequentially through 10 nm band pass filters centred at 340, 380 and 490 nm housed in computer-controlled filter wheel (Cairn Research, Kent, UK). Emitted fluorescence light was reflected through a 515 nm long-pass filter to a cooled CCD camera (Retiga, QImaging, Canada). All imaging data were collected and analysed using the Andor software (Belfast, UK). The fura-2 data were not calibrated in terms of [Ca^2+^]_*c*_ because of the uncertainty arising from the use of different calibration techniques. The fluorescent signal is quenched by Rh123 accumulation in polarised mitochondria; in response to depolarisation the fluorescence signal is dequenched; an increase in Rh123 signal therefore indicates mitochondrial depolarisation. We normalised the signals between resting level (set to 0) and a maximal signal generated in response to the uncoupler FCCP (1 μM; set to 100%).

### Data and statistical analysis

*In vivo* and *in vitro* results are shown related to healthy donors and untreated cells, respectively. All of them are expressed as mean ± standard error of the mean (SEM) in percentage. *In vitro* data were generated from a minimum of three independent replicates per experiment (n = 3) performed in different days. For *in vivo* imaging, each replicate consisted of at least 1 coverslip per condition where a number of 15–30 cells per coverslip were analysed. Statistical analysis and exponential curve fitting were performed using GraphPad Prism 6.01 (GraphPad Software, La Jolla, CA, USA) software. Grubbs outlier filter was used for all data. Statistical significance for multiple comparisons was calculated by one-way ANOVA followed by Fisher’s LSD correction. In all cases, statistical significance was set at *p* < 0.05 (*p < 0.05, **p < 0.01, ***p < 0.001; (#p < 0.05, ##p < 0.01, ###p < 0.001, ####p < 0.0001).

### Ethical approval and informed consent

All participants gave written informed consent for participation. ARRIVA guidelines for the care and use of animals were followed. The project in full was approved by the 12 de Octubre Research Institute ethical review committee. Additionally, research related to CSF samples was also approved by the 12 de Octubre Hospital, (Madrid, Spain) and Hospital de la Santa Creu i Sant Pau (Barcelona, Spain) review committees; research involving the use of choroid plexus samples from human donors was also approved by the Institute of Neuropathology Brain Bank IDIBELL-Hospital Universitari de Bellvitge (Hospitalet de Llobregat, Spain) review committee, The Netherlands Brain Bank (NBB) (Amsterdam, The Nederlands) review committee and Banco de Tejidos, Fundación CIEN (Centro de Investigación de Enfermedades Neurológicas, Madrid. Spain) review committee.

## Supplementary information

Supplementary Figure 1.

## Data Availability

The datasets generated and/or analysed during the current study are available from the corresponding author on reasonable request.
