# Supplementary material for: Annexin A5 prevents amyloid-β-induced toxicity in choroid plexus: implication for Alzheimer’s disease
Source: Sci Rep. 2020 Jun 10;10:9391. doi: 10.1038/s41598-020-66177-5 (PMC7286910; doi:10.1038/s41598-020-66177-5)
Supplement: Supplementary file 1 — Supplementary Figure 1. [file 41598_2020_66177_MOESM1_ESM.pdf]

Supplementary Information

Annexin A5 prevents amyloid-β-induced toxicity in choroid plexus: implication for Alzheimer's disease

Fernando Bartolome, Agnieszka Krzyzanowska, Macarena de la Cueva, Consuelo Pascual, Desiree Antequera, Carlos Spuch, Alberto Villarejo-Galende, Alberto Rabano, Juan Fortea, Daniel Alcolea, Alberto Lleo, Isidro Ferrer, John Hardy, Andrey Y Abramov, Eva Carro.

Supplementary Figure 1 – Full-length blots from Figures 1b, 3a and b, and 4a – d.

a – Full-length blots from Figure 1b corresponding to annexin A5 (left) and β-actin as loading control (right). In both, dashed lines show the cropped sections shown in Figure 1b.

b – Full-length blots from Figure 3 corresponding to annexin A5 (top left) and β-actin as loading control (bottom left) in cell lysates (Figure 3a). The image shows other bands corresponding to other different experimental conditions. Gel 1 and Gel 2 means that two different blots from different experimental conditions were developed at the same and the bands for the experiment described in figure 3 correspond to Gel 1. On the right, full-length blot showing annexin A5 levels from extracellular medium (Figure 3b). The image shows other bands corresponding to other different experimental conditions. In all cases, dashed lines show the cropped sections shown in Figure 3.

c - Full-length blots from Figure 4 corresponding to LC3-I and LC3-II bands (Figure 4a), p62 (Figure 4b) and β-actin (Figure 4a and b) as loading control from (left panels). On the right, full-length blots showing LC3II (Figure 4c), p62 (Figure 4d) and β-actin (Figure 4c and d) as loading control. The image shows other bands corresponding to other different conditions. In all cases, dashed lines show the cropped sections shown in Figure 4a, b, c and d.

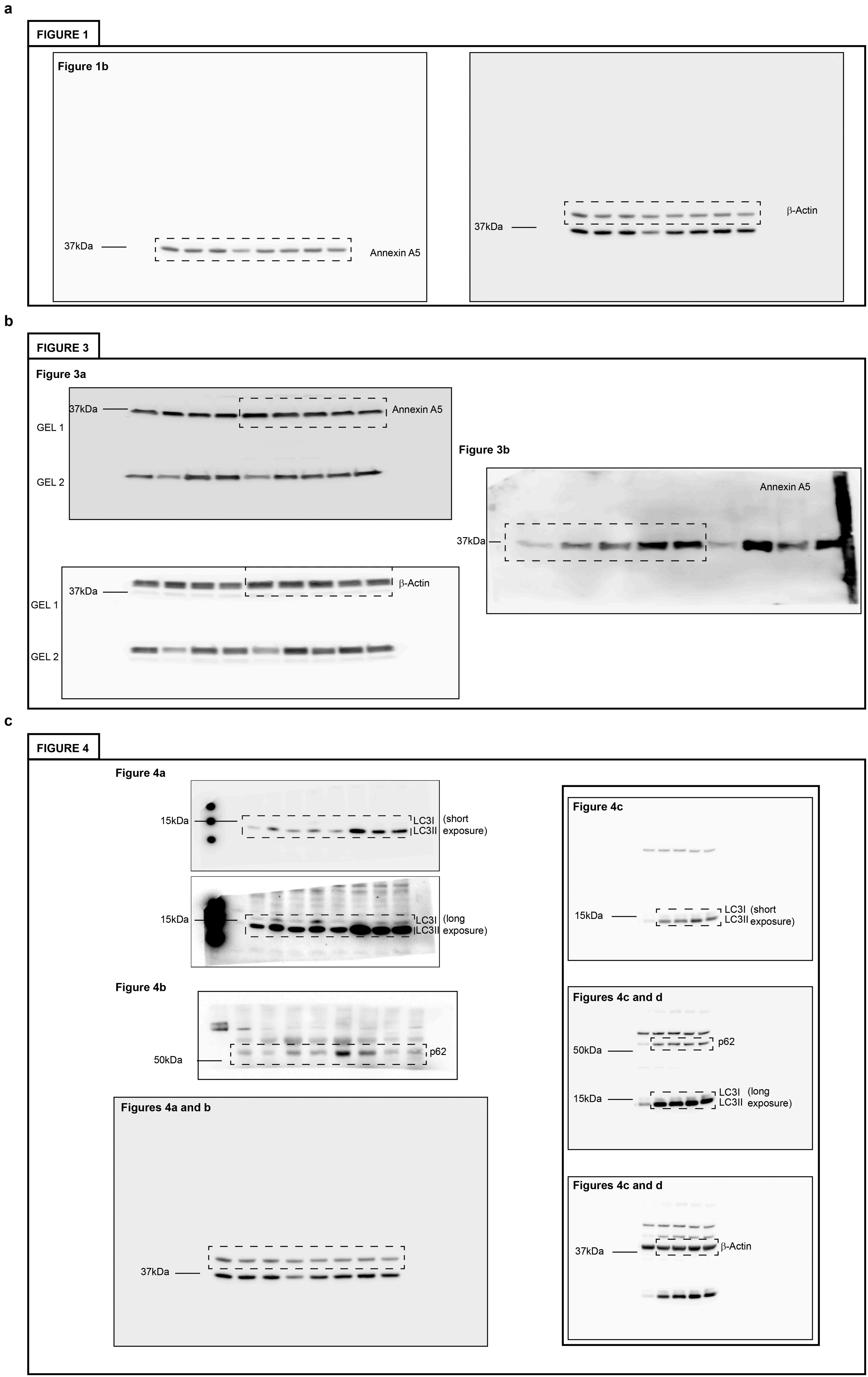
a
FIGURE 1
Figure 1b
37kDa
Annexin A5
37kDa
β-Actin
b
FIGURE 3
Figure 3a
37kDa
Annexin A5
GEL 1
GEL 2
37kDa
β-Actin
GEL 1
GEL 2
Figure 3b
Annexin A5
37kDa
c
FIGURE 4
Figure 4a
15kDa
LC3I
LC3II
(short exposure)
15kDa
LC3I
LC3II
(long exposure)
Figure 4b
50kDa
p62
Figures 4a and b
37kDa
Figure 4c
15kDa
LC3I
LC3II
(short exposure)
Figures 4c and d
50kDa
p62
15kDa
LC3I
LC3II
(long exposure)
Figures 4c and d
37kDa
β-Actin
